# Supplementary material for: Transthyretin Amyloid Cardiomyopathy: Impact of Transthyretin Amyloid Deposition in Myocardium on Cardiac Morphology and Function
Source: J Pers Med. 2022 May 13;12(5):792. doi: 10.3390/jpm12050792 (PMC9147607; doi:10.3390/jpm12050792)
Supplement: Supplementary file 1 [file jpm-12-00792-s001.zip › jpm-1655251-supplementary.pdf]

Journal of Personalized Medicine

Supplementary Materials

Text S1. Immunohistochemistry protocol for 8-hydroxy-2′-deoxyguanosine using human cardiac tissue specimens.

Human cardiac tissue specimens were fixed with 10% buffered formalin, embedded in paraffin, and sectioned at 4-μm thickness. After deparaffinization and hydration, the tissues were blocked with Tris-buffered saline with Tween-20 (TBST) containing 4% normal donkey serum (Sigma-Aldrich, St. Louis, MO, USA) and 2% bovine serum albumin for 1 h. The tissues were incubated with anti-8-hydroxy-2′-deoxyguanosine (8-OHdG) antibody (ab48508, 1:200 dilution; Abcam, Cambridge, United Kingdom) at 4°C overnight. Histofine Simple Stain Max PO (M) (424131, Nichirei, Tokyo, Japan) was used as the secondary antibody, and diaminobenzidine (Simple Stain DAB Solution, 425011, Nichirei, Tokyo, Japan) was used as a chromogenic substrate. All sections were counterstained with hematoxylin.

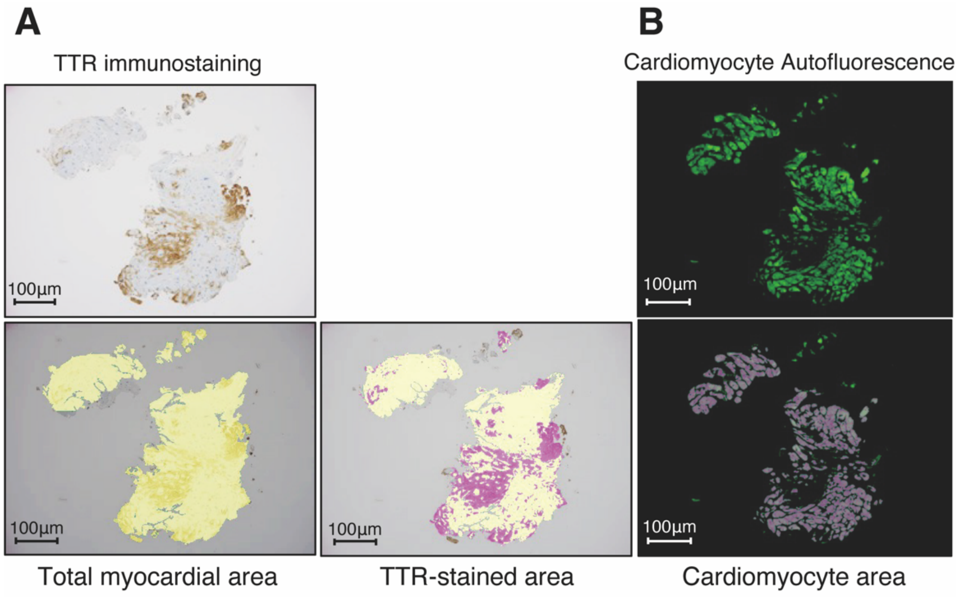

Figure S1. Images of TTR immunostaining and autofluorescence in patients with ATTR-CM. (**A**) The total myocardial area and TTR-stained area were measured using TTR immunostaining. (**B**) Cardiomyocyte autofluorescence was used to measure cardiomyocyte area. ATTR-CM, transthyretin amyloid cardiomyopathy; TTR, transthyretin.

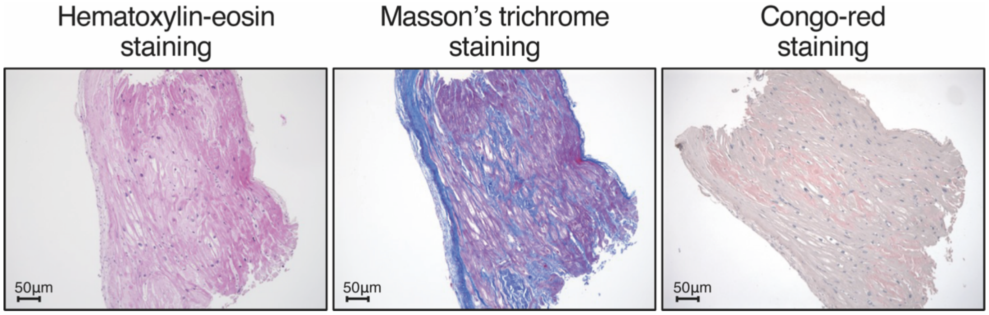

Figure S2. General staining of endomyocardial biopsy specimens of patients with ATTR-CM. Endomyocardial biopsy specimens of patients with ATTR-CM were stained using hematoxylin-eosin, Masson's trichrome, and Congo red staining. ATTR-CM, transthyretin amyloid cardiomyopathy.

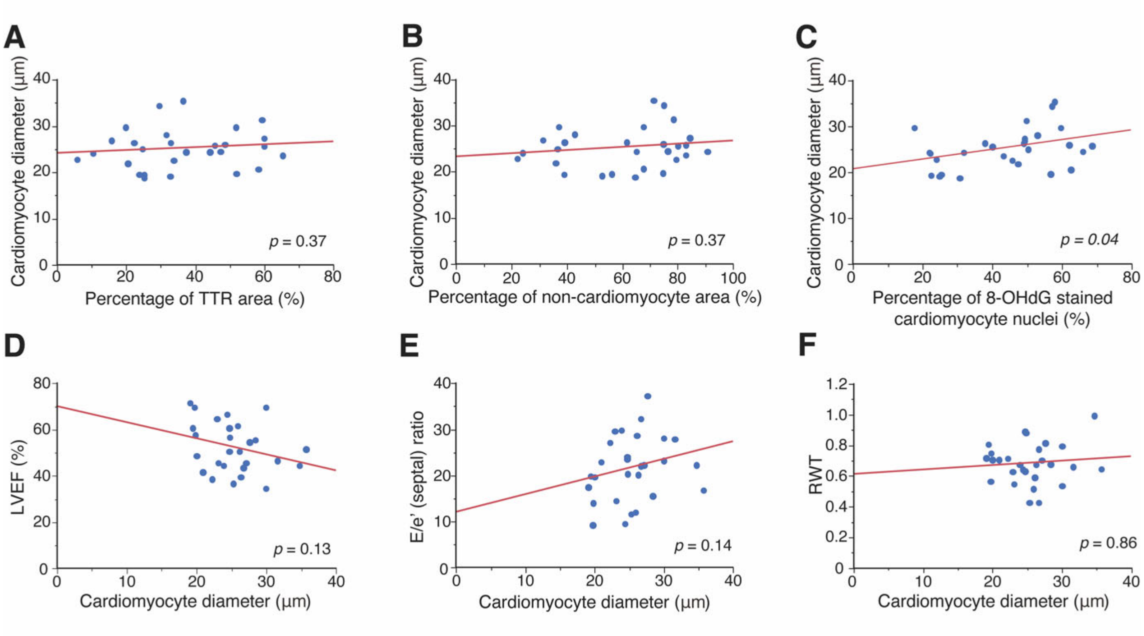

Figure S3. Association of cardiomyocyte diameter and pathological and echocardiographic features in patients with ATTR-CM. The relationship between cardiomyocyte diameter and (A) percentage of TTR area, (B) percentage of non-cardiomyocyte area, (C) percentage of 8-OHdG stained cardiomyocyte nuclei, (D) LVEF, (E) E/e' (septal) ratio, and (F) RWT. ATTR-CM, transthyretin amyloid cardiomyopathy; E/e' (septal) ratio, early transmitral filling velocity (E)/peak early diastolic velocity of septal mitral annulus (e') ratio; LVEF, left ventricular ejection fraction; 8-OHdG, 8-hydroxy-2'deoxyguanosine; RWT, relative wall thickness; TTR, transthyretin.
